# A Comparison of Varying Golden Proportion on Smile Aesthetic Perception Between Patients, Orthodontists and Restorative Dentists

**DOI:** 10.1155/ijod/9077108

**Published:** 2026-06-11

**Authors:** Maryam Saeed, Nijah Ahmed, Tania Arshad Siddiqui, Nadia Aman, Shoaib Rahim, Mansoor Khan

**Affiliations:** ^1^ Department of Orthodontics, Foundation University Islamabad, Islamabad, Pakistan, fui.edu.pk; ^2^ Department of Operative Dentistry, Foundation University Islamabad, Islamabad, Pakistan, fui.edu.pk; ^3^ Department of Prosthodontics, Foundation University Islamabad, Islamabad, Pakistan, fui.edu.pk

**Keywords:** aesthetic treatment, golden proportion, interdisciplinary collaboration, orthodontics, restorative dentistry

## Abstract

**Objective:**

The study aimed to assess how variations in the golden proportion (GP) influence aesthetic perception among three groups: orthodontic patients, orthodontists and restorative dentists. It sought to determine a range of aesthetic tolerance for lateral incisors and canines, addressing gaps in previous literature.

**Method and Materials:**

Our cross‐sectional study included 128 participants (orthodontic patients and clinicians). Standardised smile photographs were digitally altered from a range of 52%–77% at 5% intervals. Participants rated photos on a five‐point Likert scale. The range was determined by analysing scores using the interquartile range (IQR), focusing on photographs with a median ≥3 and outliers were then removed. Kruskal–Wallis and Mann–Whitney *U* tests compared groups, while Spearman correlation evaluated gender and experience.

**Results:**

Patients accepted a broader range of GP (52%–77%) compared to orthodontists (62%–67%), operative dentists (57%–72%) and prosthodontists (62%–72%). Between‐group differences were significant for the majority of photographs (*p*  < 0.05), with male photographs reaching high significance (*p* < 0.001). Post hoc analysis confirmed consistent differences between patients and professionals (*p* < 0.001), while fewer differences existed among professional groups, showing weak to moderate agreement (rb = 0.257–0.396). Gender (*p* > 0.05, except for two images: *p* = 0.013, *R* = 0.255) and clinical experience (*p* > 0.05) did not significantly influence aesthetic preferences.

**Conclusions:**

The study concludes that the GP is more applicable as a range rather than a fixed value. Patients showed a wider aesthetic preference than clinicians. The patients’ perceptions were closer to restorative dentists than orthodontists (*p* < 0.001), likely reflecting differing clinical objectives. The inter‐professional agreement observed supports the need for a multidisciplinary approach in aesthetic treatment planning.

**Clinical Significance:**

The clinical significance of the study lies in its potential to improve treatment outcomes in both aesthetic dentistry and orthodontics. The study highlights the need for a flexible, range‐based approach while using the GP to achieve diverse aesthetic demands. The importance of interdisciplinary collaboration between restorative dentists and orthodontists in procedures such as space closures and teeth remodelling is emphasised, ultimately improving aesthetic and functional outcomes. It also acknowledges the variability in aesthetic perception among different cultural and ethnic backgrounds.

## 1. Introduction

Achieving an attractive, well‐balanced smile has become the paramount objective of modern orthodontic treatment [1]. Researchers have increasingly focused on comprehending the factors contributing to smile aesthetics. Many guidelines, including the golden proportion (GP) have been proposed for designing symmetrical and aesthetic smiles. The GP principle proposed by Levin [2] is the ratio between two adjacent teeth where the lateral incisor is 62% of the central incisor and the canine is 62% of the lateral incisor. However, this ratio is not frequently observed in natural dentition despite its historical prominence. Recent studies question the GP’s applicability to diverse populations [3].

Preston [4] states that the GP is not always found in the natural dentition, suggesting a more flexible approach. The recurrent aesthetic dental (RED) theory [5] supports this by arguing that aesthetics is dynamic and should consider individual features over static formulas. This evolving understanding emphasises the clinician’s need to create smiles that are personalised for patients.

Cultural and ethnic variations play a significant role in the perception of the GP. Researchers indicate that the GP’s applicability varies across populations [6]. For instance, the GP was not detected in the Turkish population [7], whereas it was preferred as an aesthetic guide in the Bangladeshi population [8].

In Pakistan, it was considered second best for normal‐sized teeth but ideal for teeth with increased crown height [9]. Rosentiel et al. [10] found the same result in their study. No single aesthetic formula was found to be universally applicable, with the GP emerging as the second most preferred [11]. Gender also influences aesthetic perception, with some research showing its significant influence on aesthetics [12, 13].

Recent studies show that the GP may hold validity in some aspects [14]. Notably, studies have suggested that it may apply more as a range rather than a strict value [15–18] and that slightly modified GP values may be more representative and relevant in smile design [19].

Moreover, a slight divergence from ideal proportions often goes unnoticed by laypersons, general dentists, and orthodontists, reinforcing that small variations are aesthetically acceptable [20]. Notably, the GP is observed more commonly between central incisors and lateral incisors than between lateral incisors and the canines [21]. A broader display of canines is preferred in the digital setting in some studies [22], further highlighting the need to consider individual tooth morphology while designing a smile.

Patient perception of aesthetics has been emphasised while formulating treatment plans, as studies reveal notable differences in aesthetic perception between patients and clinicians [23, 24]. Patients usually exhibit a broader, flexible range of acceptance [25], while orthodontists were the most critical regarding smile aesthetics [24, 26], preferring GPs for central incisors [27]. Younger patients usually demonstrate greater sensitivity to aesthetics [28, 29]. Interestingly, a heightened awareness of aesthetic perception is found in orthodontic patients. Their perception of aesthetics is improved during the treatment process [30], making their evaluations more reliable.

In terms of clinical results, studies show high aesthetic stability and patient satisfaction when the GP is applied in smile makeovers [31], highlighting its significance. The relative influence of different smile parameters on attractiveness has also been studied, with the width‐to‐length proportion of anterior teeth classified as a medium‐impact parameter by professionals [32]. Additionally, recent studies have begun to take into account facial characteristics like inner canthal distance and interpupillary width in assessing the GP [33, 34], thereby enhancing the personalisation of smile design.

A notable preference for female pictures is seen when assessing smile attractiveness [35, 36]. The varying effect of buccal corridors on smile aesthetics has been seen [37, 38] while whiter teeth are perceived as aesthetically pleasing, regardless of gender [39]. At the same time, some studies show that years of clinical experience refine aesthetic perception [40]. Others, however, show the minimal impact of clinical experience on aesthetic refinement [41, 42].

## 2. Aim

This study aimed to assess how variations in the GP influence aesthetic perception among three groups: orthodontic patients, orthodontists and restorative dentists. The study sought to determine a range of aesthetic tolerance by examining how orthodontic patients, orthodontists and restorative dentists perceive aesthetic judgements. It employed a controlled setup with standardised smile images and uniform presentation conditions to ensure that any differences in perception directly resulted from variations in the GP.

## 3. Hypothesis

• The traditional GP will receive higher aesthetic ratings compared to the altered proportions by all groups.
• Significant differences in aesthetic perception will be present between orthodontic patients, orthodontists and restorative dentists on altered GPs.
• Years of clinical experience will significantly affect the aesthetic perception of altered GPs in dental professionals.

## 4. Materials and Methods

A sample of 128 participants (selected via criterion‐based consecutive purposive sampling), 34 orthodontic patients, 32 orthodontists, 32 prosthodontists and 30 operative dentists of Pakistani origin were selected based on a pilot study [17]. A sample size of 21 (per group) was obtained with an effect size of 0.59 to detect a difference in the actual and apparent width of the lateral incisor with a power of 80% and a level of significance at 0.05. Thus, the final required size was 84. To improve the precision of estimates, better balance the four groups, and allow for potential exclusions, the sample size was increased to 128. Using the achieved sample, post hoc calculations showed a statistical power of 98.87% for the main outcome. Patients, 13–30 years old, presenting to the orthodontic OPD in Pakistan, with at least a 1 year history of orthodontic treatment and no learning and comprehension disabilities were considered. The clinicians who had cleared the FCPS Part II/MDS/equivalent post‐graduate exit exam were included. All participants gave informed consent, and the study protocol was approved by the ethics review committee, FUCD&H (FF/FUCD/632/ERC/74).

## 5. Data Collection

This cross‐sectional study was carried out in dental hospitals in Pakistan over a period of 4 months (14th May to 20th August 2024). Six frontal‐posed smile photographs of female and male individuals (not part of the sample population) with no history of orthodontic treatment were taken using a Canon EOS 1100D DSLR with a 50–70 mm macro lens at a fixed height, ~1.2 m from the subject. Subjects were seated in a natural head position with the Frankfort horizontal plane parallel to the floor and the midsagittal plane perpendicular to the camera. Identical lighting and camera settings (f/16, 1/125 s, ISO 100) were used, and framing was limited to the lips and maxillary anterior teeth.

**Figure 1:**
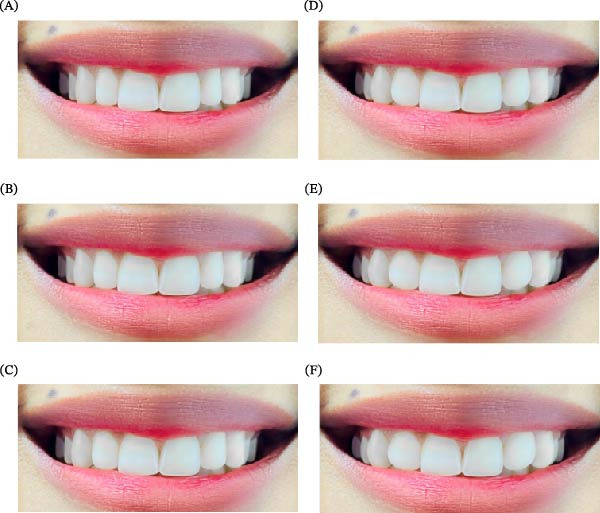
Smiles after manipulation of female maxillary lateral incisor width percentages: (A) 52%; (B) 57%; (C) 62%; (D) 67%; (E) 72%; (F) 77%.

**Figure 2:**
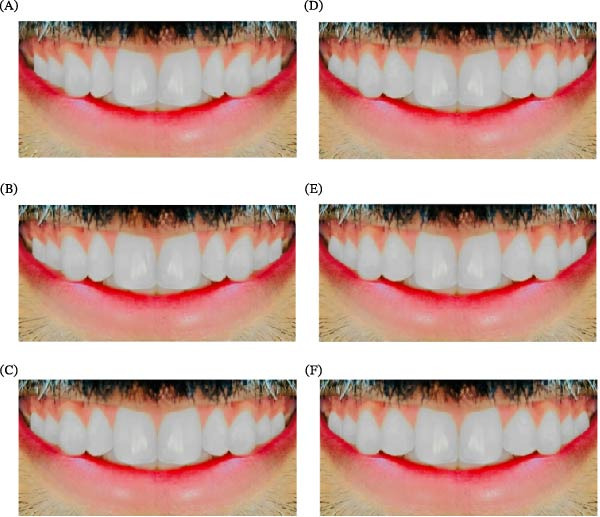
Smiles after manipulation of male maxillary lateral incisor width percentages: (A) 52%; (B) 57%; (C) 62%; (D) 67%; (E) 72%; (F) 77%.

**Figure 3:**
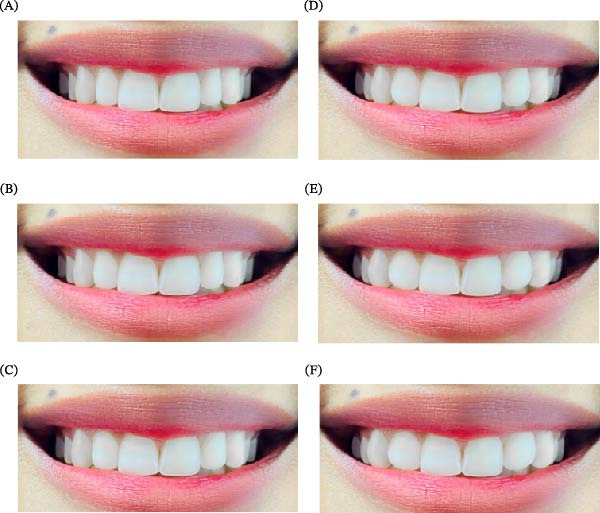
Smiles after manipulation of female maxillary canine width percentages: (A) 52%; (B) 57%; (C) 62%; (D) 67%; (E) 72%; (F) 77%.

**Figure 4:**
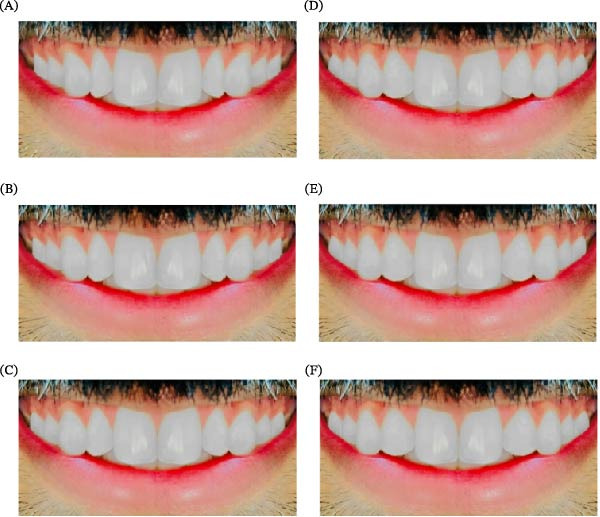
Smiles after manipulation of male maxillary canine width percentages: (A) 52%; (B) 57%; (C) 62%; (D) 67%; (E) 72%; (F) 77%.

The photos were arranged with a 15 s viewing time in a PowerPoint presentation and shown to a panel of two orthodontists and an operative dentist from the Foundation University College of Dentistry and Hospital. They were given a score on a 100 mm visual analogue scale based on perceived aesthetics. The top‐scoring photos were selected, a male and a female, to reduce selection bias. They were digitally altered using Adobe Photoshop at 5% [18] intervals over a range of 52%, 57%, 62%, 67%, 72% and 77% for the central and lateral incisor and lateral incisor and canine (Figures 1–4). To eliminate confounding factors, tooth shade correction [39] was digitally standardised across both male and female photographs via Adobe Photoshop’s hue/saturation and brightness/contrast adjustment tools. The male subject’s photo was split, inverted and stitched to address the original asymmetry. Overall, 4 sets were formed, with each set containing 6 pictures, forming a total of 24 photos. These were randomised via an online random sequence generator (random.org) to reduce observer bias.

A PowerPoint slide show with the 24 altered pictures, each with a 10 s display time, was shown to the participants, and their responses were recorded. All participants were asked to rank the photographs on a five‐point Likert scale. The questionnaires were additionally translated into Urdu with the help of an editor for the patients to reduce language comprehension bias.

Clinicians were not always easily available in dental hospitals due to a clash in the data collectors’ and the clinicians’ schedules; therefore, to maximise reach, an online version of the form (Google Form), with an incorporated timer, was created and sent to specialists. To maintain uniformity, the item content, order response options, scoring procedures and time were kept identical across all formats, thereby maintaining conceptual equivalence of measurement. The online version recorded the names of the participants also, and all online and on‐paper responses were vetted carefully to avoid double entries.

## 6. Statistical Analysis

Statistical analysis was run on IBM SPSS Statistics for Windows, Version 23.0. Descriptive statistics, that is, mean, median and frequency for age and gender, were determined. As the data was not normally distributed, non‐parametric tests were employed. Ratings among the four groups (patients, orthodontists, operative dentists and prosthodontists) were compared via the Kruskal–Wallis test and further comparison was done between the two groups via Mann–Whitney *U* analysis.

In order to avoid sparse categories and to reflect a clinically meaningful distinction in aesthetic judgement, the 5‐point scale (1 = very unpleasant, 2 = unpleasant, 3 = acceptable, 4 = pleasant, 5 = very pleasant) was collapsed into two categories: “unacceptable” (scores 1–2) and “acceptable” (scores 3–5) [43]. The mean and median for each group were calculated and compared. The kurtosis and skewness confirmed the data as mildly skewed and platykurtic; hence, both mean and median were relevant descriptive parameters.

The interquartile range (IQR) was calculated for medians of selected photographs. Boxplots were generated to visualise the distribution of the ranking. Outliers of the data were removed using the IQR to determine the range. The degree of association between years of experience and gender‐influenced results was calculated by Spearman’s correlation. Finally, the degree of agreement for the professional groups with statistically significant Mann–Whitney *U* results was calculated using rank‐biserial correlation analysis. A *p*‐value ≤ 0.05 was considered statistically significant.

## 7. Results

The characteristics of participants are summarised in Table 1. The study included 128 participants. The mean ages of dental professionals and orthodontic patients were 38.60 ± 6.27 years and 19.82 ± 4.32 years, respectively. The dental professionals had varied years of experience, with 26.6% having less than 1 year, 36.7% having 1–5 years, 19.5% having 6–10 years, 11.7% having 11–15 years and 5.5% having 16–20 years.

**Table 1:** Descriptive statistics of the sample.

Total	128
Gender	
Male	44 (34.4%)
Female	84 (65.6%)
Group	
Orthodontic patients	34
Orthodontists	32
Operative dentists	30
Prosthodontists	32
Mean age	
Dental professionals	38.60 ± 6.27 years
Orthodontic patients	19.82 ± 4.32 years
Mean years of experience (dental professionals)	6.54 ± 4.67 years

**Figure 5:**
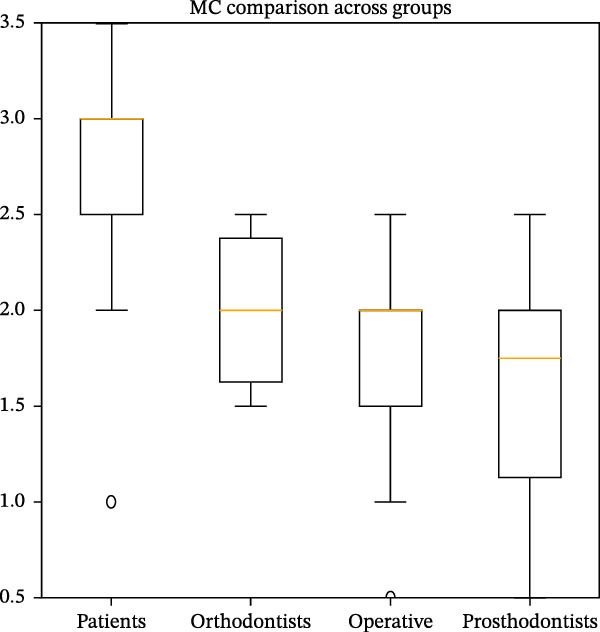
Box plot 1 showing distribution of male patients’ rating across different canine width percentages.

**Figure 6:**
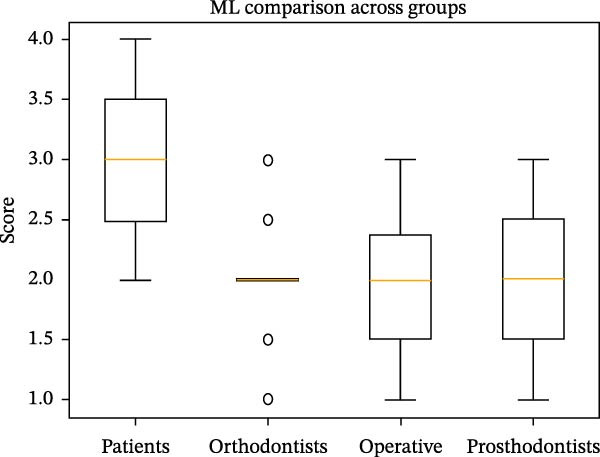
Box plot 2 showing distribution of male patients’ rating across different lateral incisor width percentages.

**Figure 7:**
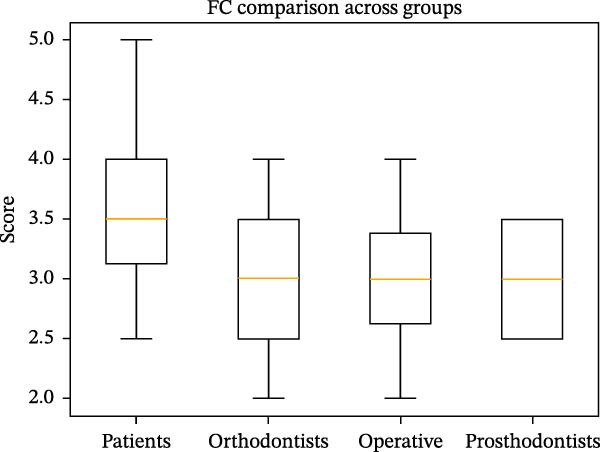
Box plot 3 showing distribution of female patients’ rating across different canine width percentages.

**Figure 8:**
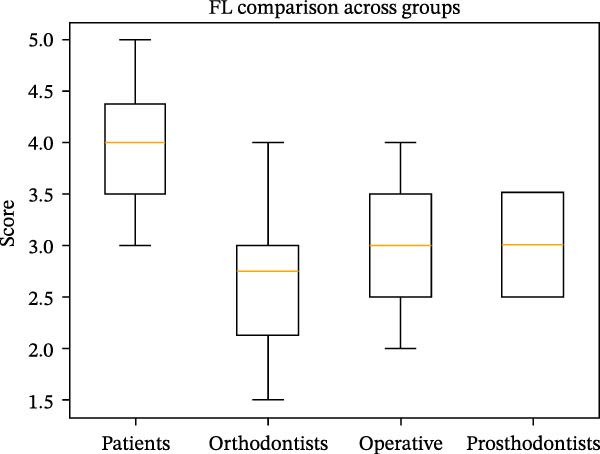
Box plot 4 showing distribution of female patients’ rating across different female lateral width percentages.

The median scores and IQRs for all photographs across the four groups are presented in Table 2, with Kruskal–Wallis *p*‐values for each comparison. Among patients, the median ratings ranged from 2 to 4. The 77% and 72% proportions received the highest median scores for canines, while 52% and 67% were rated the highest for lateral incisors. For dental professionals, orthodontists showed median scores of 2–3, operative dentists 1–3 and prosthodontists 1.5–3. These distributions are visualised in Figures 5–8.

**Table 2:** Comparison of smile attractiveness for golden proportion among the groups.

		Patient (34)		Orthodontist (32)		Operative (30)		Prosthodontist (32)		*p*‐Value
Variable		Median	IQR	Median	IQR	Median	IQR	Median	IQR	
ML	52	3	1	2	0	2	1	2	2	<0.001 ^∗∗∗^
	57	3	2	2	1	2	2	2	1	<0.001 ^∗∗∗^
	62	3	1	2	0	2	1	2	1	<0.001 ^∗∗∗^
	67	3	2	2	2	2	1	2	1	0.003 ^∗^
	72	3	2	2	0	1.5	1	2	1	0.001 ^∗∗^
	77	3	1	2	0	2	1	2	1	<0.001 ^∗∗∗^
FL	52	4	2	2.5	1	3	1	3	1	0.001 ^∗∗^
	57	4	2	3	2	3	1	3	1	0.66
	62	4	1	3	1	3	1	3	1	0.007 ^∗^
	67	4	1	3	2	3	1	3	1	0.068
	72	4	1	3	1	3	2	3	1	0.020 ^∗^
	77	3.5	1	2	1	3	1	3	1	<0.001 ^∗∗∗^
MC	52	2	2	2	1	1	1	1	1	<0.001 ^∗∗∗^
	57	3	1	2	1	2	1	1	1	<0.001 ^∗∗∗^
	62	3	1	2	1	2	0	2	0	<0.001 ^∗∗∗^
	67	3	1	2	0	2	1	1.5	1	<0.001 ^∗∗∗^
	72	3	1	2	1	2	1	2	1	0.001 ^∗∗^
	77	3	0	2	1	2	0	2	0	<0.001 ^∗∗∗^
FC	52	4	1	3	2	3	1	3	1	0.056
	57	3.5	1	3	1	3	1	3	1	0.040 ^∗^
	62	3	1	3	1	3	0	3	1	0.313
	67	3.5	1	3	1	3	1	3	1	0.277
	72	4	2	3	1	3	1	3	1	0.065
	77	4	1	3	1	3	2	3	1	0.002 ^∗^

*Note: N*‐128. Kruskal–Wallis test. *p* value: 0.05 ^∗^, ≤0.001 ^∗∗^, <0.001 ^∗∗∗^.

Abbreviations: C, canine; F, female; L, lateral; M, male.

As per the methodology, photographs with median scores ≥3 were classified as acceptable, and those below 3 as not acceptable (Table 2). The IQR was used for the refinement of the GP range by removing outliers, leaving 75% of the data in the acceptable range. The resulting ranges are presented in Table 3.

**Table 3:** Range of acceptable golden proportion among the groups.

Group	Lateral incisor range (before refining)	Lateral incisor range (after refining)	Canine range (before refining)	Canine range (after refining)
Patients	52%–77%	52%–77%	52%–77%	52%–77%
Orthodontists	57%–62%	62%–67%	52%–72%	62%–67%
Operative dentists	57%–72%	57%–72%	57%–77%	57%–72%
Prosthodontists	52%–67%	62%–67%	52%–72%	62%–72%

Patients accepted a GP range of 52%–77% for both lateral incisors and canines. Orthodontists preferred a narrower range of 62%–67% for both. Operative dentists preferred 57%–72%, and prosthodontists favoured 62%–72%. The 62%–67% proportion was most agreed upon among all dental professionals for both lateral incisors and canines.

Kruskal–Wallis analysis demonstrated significant between‐group differences for the majority of width ratios, with male lateral incisor photographs reaching high significance (ML52%, ML57%, ML62%, ML77%; all *p*  < 0.001) and male canine photographs showing similar patterns (MC57%, MC62%, MC67%, MC77%; all *p*  < 0.001). Female lateral incisor photographs showed significant differences at 52% (*p* = 0.001), 62% (*p* = 0.007), 72% (*p* = 0.020) and 77% (*p*  < 0.001). Female canine photographs demonstrated fewer significant differences, with only FC57% (*p* = 0.040) and FC77% (*p* = 0.002) reaching significance (Table 2).

Post hoc Mann–Whitney *U* analysis (Table 4) revealed that the most consistent differences were between patients and dental professionals (*p*  < 0.001 for 18 of 24 photographs). Fewer significant differences were found among professional groups. Exceptions included orthodontists versus prosthodontists in picture 5, FC77% (*p* = 0.05, rb = 0.257), picture 9, FC62% (*p* = 0.028, rb = 0.297), picture 14, MC72% (*p* = 0.030, rb = 0.29) and picture 16, FL52% (*p* = 0.004, rb = 0.396). A significant difference was observed between operative dentists and prosthodontists in picture 13, FL62% (*p* = 0.011, rb = 0.340), and between orthodontists and operative dentists in picture 20, ML72% (*p* = 0.028, rb = 0.299).

**Table 4:** Intergroup comparison for differences in aesthetic perception for golden proportion.

Picture number	Pt/ortho	Pt/oper	Pt/prostho	Ortho/oper	Ortho/prostho	Oper/prostho
1 (MC 62%)	0.002 ^∗^	<0.001 ^∗∗∗^	<0.001 ^∗∗^	0.407	0.266	0.728
2 (FC 72%)	<0.001 ^∗∗∗^	0.013 ^∗^	0.004 ^∗^	0.125	0.124	0.921
3 (MC 77%)	<0.001 ^∗∗∗^	<0.001 ^∗∗∗^	<0.001 ^∗∗∗^	0.248	0.479	0.65
4 (FL 67%)	<0.001 ^∗∗∗^	<0.001 ^∗∗∗^	<0.001 ^∗∗∗^	0.55	0.42	0.85
5 (FC 77%)	<0.001 ^∗∗∗^	<0.001 ^∗∗∗^	<0.001 ^∗∗∗^	0.739	0.05 ^∗^ rb = 0.257	0.243
6 (ML 52%)	<0.001 ^∗∗∗^	<0.001 ^∗∗∗^	<0.001 ^∗∗∗^	0.093	0.928	0.250
7 (MC 57%)	<0.001 ^∗∗∗^	<0.001 ^∗∗∗^	<0.001 ^∗∗∗^	0.407	0.064	0.349
8 (FC 52%)	0.014 ^∗^	0.001 ^∗∗^	0.06	0.235	0.566	0.077
9 (FC 62%)	0.008 ^∗^	0.033 ^∗^	0.643	0.572	0.028 ^∗^ rb = 0.297	0.087
10 (ML 67%)	0.004	<0.001 ^∗∗∗^	<0.001 ^∗∗∗^	0.406	0.561	0.771
11 (ML 57%)	<0.001 ^∗∗∗^	<0.001 ^∗∗∗^	<0.001 ^∗∗∗^	0.735	0.512	0.790
12 (FL 57%)	0.002 ^∗^	0.001 ^∗∗^	0.003 ^∗^	0.738	0.74	0.504
13 (FL 62%)	0.001 ^∗∗^	<0.001 ^∗∗∗^	0.022 ^∗^	0.256	0.102	0.011 ^∗^ rb = 0.340
14 (MC 72%)	0.047	<0.001 ^∗∗∗^	<0.001 ^∗∗∗^	0.187	0.030 ^∗^ rb = 0.29	0.566
15 (FL 72%)	<0.001 ^∗∗∗^	0.003 ^∗^	0.004 ^∗^	0.129	0.472	0.395
16 (FL 52%)	<0.001 ^∗∗∗^	<0.001 ^∗∗∗^	0.002 ^∗^	0.103	0.004 ^∗^ rb = 0.396	0.204
17 (ML 77%)	<0.001 ^∗∗∗^	0.002	<0.001 ^∗∗∗^	0.366	0.396	0.862
18 (FC 57%)	<0.001 ^∗∗∗^	0.02	0.024 ^∗^	0.263	0.142	0.824
19 (FL 77%)	0.001 ^∗∗^	0.001 ^∗∗^	0.004 ^∗^	0.192	0.647	0.442
20 (ML 72%)	0.007	<0.001 ^∗∗∗^	0.002	0.028 ^∗^ rb = 0.299	0.376	0.184
21 (FC 67%)	0.068	0.111	0.657	0.851	0.131	0.174
22 (MC 52%)	0.116	0.012	0.20	0.260	0.335	0.898
23 (ML 62%)	0.001 ^∗∗^	<0.001 ^∗∗∗^	<0.001 ^∗∗∗^	0.313	0.134	0.607
24 (MC 67%)	0.002	0.001 ^∗∗^	<0.001 ^∗∗∗^	0.357	0.065	0.419

*Note: N*‐128. Mann–Whitney *U* test. *p* value: 0.05 ^∗^, ≤0.001 ^∗∗^, <0.001 ^∗∗∗^.

Abbreviations: C, canine; F, female; L, lateral; M, male; rb, rank biserial correlation.

Analysis of rank‐biserial correlations for pictures with significant inter‐professional differences showed weak to moderate positive agreement (rb = 0.257–0.396), suggesting that despite differences in specific proportions, professional groups maintained a degree of consensus in aesthetic preferences.

Spearman correlation analysis (Table 5) showed that gender had no significant influence on aesthetic preferences (*p*  > 0.05) except for a weak positive correlation in two images: ML52% (*p* = 0.013, *R* = 0.255) and MC67% (*p* = 0.013, *R* = 0.255). No significant effect of years of clinical experience was found on GP preferences (*p*  > 0.05).

**Table 5:** Correlation between years of experience and gender of professionals with acceptable range of golden proportion.

Picture number	Gender		Years of experience		
	*R*	*p*‐Value	*r*	*p*‐Value	
1 (MC 62%)	0.091	0.384	−0.059	0.569	
2 (FC 72%)	0.170	0.101	−0.025	0.808	
3 (MC 77%)	−0.035	0.736	−0.033	0.751	
4 (FL 67%)	0.105	0.312	0.058	0.577	
5 (FC 77%)	0.041	0.694	0.130	0.212	
6 (ML 52%)	0.255 ^∗^	0.013	−0.097	0.354	
7 (MC 57%)	0.027	0.799	−0.049	0.637	
8 (FC 52%)	−0.002	0.984	0.102	0.330	
9 (FC 62%)	−0.028	0.790	0.035	0.739	
10 (ML 67%)	−0.002	0.983	−0.014	0.895	
11 (ML 57%)	−0.092	0.377	−0.013	0.898	
12 (FL 57%)	0.041	0.694	0.024	0.817	
13 (FL 62%)	0.143	0.170	0.029	0.780	
14 (MC 72%)	−0.022	0.833	−0.068	0.514	
15 (FL 72%)	0.064	0.543	0.008	0.936	
16 (FL 52%)	0.167	0.108	0.072	0.493	
17 (ML 77%)	0.109	0.296	−0.097	0.351	
18 (FC 57%)	0.002	0.983	−0.117	0.262	
19 (FL 77%)	−0.121	0.245	0.083	0.427	
20 (ML 72%)	0.085	0.417	−0.061	0.558	
21 (FC 67%)	0.083	0.429	−0.036	0.728	
22 (MC 52%)	−0.083	0.424	0.044	0.673	
23 (ML 62%)	0.125	0.229	−0.091	0.381	
24 (MC 67%)	0.255 ^∗^	0.013	−0.165	0.112	
Spearman			Correlation		
>0.70			Very strong relationship		
0.40–0.69			Strong relationship		
0.30–0.39			Moderate relationship		
0.20–0.29			Weak relationship		
0.01–0.19			No or negligible relationship		

*Note: N*‐94. Spearman correlation coefficient. *p* value 0.05 ^∗^, ≤0.001 ^∗∗^, <0.001 ^∗∗∗^.

Abbreviations: C, canine; F, female; L, lateral; M, male.

## 8. Discussion

Previous literature reveals varying results regarding acceptability, existence in a natural population, and applicability of all aesthetic mathematical formulas since factors such as overall facial proportion, differing ethnicity, gender and general asymmetry within teeth influence the attractiveness of a smile. Hence, there is a need to determine a range, with preexisting concepts being guiding stones within specific populations.

Our first hypothesis was rejected. This conclusion was supported by the fact that the patients preferred a broader range of GP (52%–77%) for both lateral incisors and canines. They rated 52% and 67% GP highest for the lateral incisors and 72% and 77% GP for the canines. The dental professionals also showed varied preferences. Orthodontists preferred a narrow range of 62%–67% GP for both lateral incisors and canines. Operative dentists showed preference for 57%–72% GP, followed by prosthodontists favouring 62%–72% GP for both lateral incisors and canines. The results of our study were in agreement with the recent studies that questioned the universal applicability of traditional GP. Our study also favours Preston [4] and RED [5] in the fact that aesthetics are dynamic and that the individual features should be considered over the static formula. Our result was consistent with recent studies showing that GP functions more as a range rather than a value [15–18] and that slightly modified GP values are more relevant in smile designing [19]. In addition, small variations from ideal standards often go unnoticed, thus suggesting that aesthetic satisfaction is not necessarily dependent on strict adherence to traditional GP.

Our second hypothesis was accepted. Significant differences were observed among all groups (*p*  < 0.05) for all width ratios. The patients preferred a broader range of GP (52%–77 %), while a narrower range was preferred by orthodontists (62%–67%). This result is well aligned with previous investigations, where Florez et al. [24] found notable differences in aesthetic preferences between patients and clinicians and Ker et al. [25], who stated that patients exhibited a broader, flexible range of acceptance. This is attributed to higher aesthetic preferences and more objective assessments among clinicians in order to achieve good functional occlusion. Hence, clinicians had a narrower range of GP than that of patients.

Our study found no effect of years of experience on clinicians’ aesthetic perceptions. Therefore, our third hypothesis was rejected. However, the authors note that 63% of the specialists in the sample had less than 5 years of experience, which may have impacted the outcome. Our findings were contrary to a study by Al‐Saleh et al. [40], stating that heightened awareness of aesthetics was found in more experienced professionals. Our findings aligned with Ashwini et al. [41] and Rotundo et al. [42], who found no effect of experience on clinicians’ aesthetic perceptions.

Recent systemic reviews and meta‐analyses have questioned the existence of GP and also emphasised the need for flexible aesthetic guidelines [3, 15]. Our findings align with previous literature as early as Preston’s [4], who proposed a 66% lateral incisor to central ratio, and Ward [5], who suggested that the width should remain a consistent 70% across the maxillary anterior. Bukhary et al. [18] digitally modified the width of lateral incisors by 5% increments, similar to our study, and landed on the same conclusion of 67% apparent width of lateral incisors being found most appealing (*p* = 0.00001). Wolfart et al. [16] modified the perceived width of lateral incisors by 10% increments. Patients and medical students accepted photos in the range of 50%–74%, whereas dentists favoured 56%–68%. Haerian et al. [20] altered maxillary lateral incisor width by 52%–72%. About 67% was the most preferred by orthodontists, general dental practitioners and laypeople. Ker et al. [25] in their computer‐based study, allowed 243 laypeople to modify the width of lateral incisors by 0.18 mm increments by moving a slide bar. A 53%–76% range was deemed the acceptable range, with a 72% central to lateral incisor width being the most favoured. Previous studies comparing the aesthetic perception of laypeople with or without hypodontia and dentists showed all groups favouring 67%, 72% and 77% proportions for lateral incisors [18, 23].

A 2023 narrative literature [6] review found no strict aesthetic formula, including GP and RED, that fits all populations. Asians have longer teeth as compared to Caucasians, who had a greater W/L ratio instead, which is wider shorter teeth. SF Rosenstiel [10] showed that people only preferred GP for teeth with increased crown height. This was further supported by 2024 research conducted in Lahore, Pakistan [9], where GP was preferred in teeth with increased crown height by dentists. A 2023 study comparing the occurrence of GP vs RED showed GP occurring in 66.7% of the Bangladeshi population [8]. Similar results were seen in a 2010 study in the Pakistani population, which concluded that Levin’s Phi dental grid could be used to predict GP in 63% of the Pakistani population [17].

Our study did not find any significant effect of gender on aesthetic choices as supported by previous studies [6, 16, 20, 22, 25, 44] except for a weak positive correlation in two images: male lateral incisor 52% (*p* = 0.013, *R* = 0.255) and male canine 67% (*p* = 0.013, *R* = 0.255). Both images fell in the below three mean group (unpleasant). This is contrary to a study by Torul and Omezli [12], which found that gender significantly influences aesthetic judgement. Interestingly, our study also showed that female photographs were rated higher consistently, possibly due to the buccal corridor difference between the male and female photos, which aligns with previous studies [35, 36]. There is conflicting evidence regarding buccal corridors across different populations [35, 37, 38]; thus, this factor warrants cautious interpretation.

Notably, our study established an acceptable GP range for canines (52%–77%), with 77% being the most acceptable (greater canine show). Previous studies showed a lack of GP range determination or canines as they were found to be greatly affected by gender [7]. Honorato et al. [27] discovered no association between self‐perception of dental attractiveness and GP of canines among laypeople and orthodontic patients based on the Oral Aesthetic Subjective Impact Scale. However, this study analysed dental cast photos that deviate from natural‐looking smiles. GP was observed more in central and lateral incisors as compared to lateral incisors and canines [26]. The canine show had greater visibility than that proposed by Levin and Snow as observed by Lucchi et al. [14]. A 2023 meta‐analysis and systematic review [22] also found no consensus regarding the width of the canine, with some studies preferring a broader display of the canine in a digital setting. It also emphasised the urgent need for more extensive studies. Our study thus established a range for canines and provided a clinically relevant reference that was previously missing in the literature.

Young patients are more attuned to aesthetic perception [22, 28, 29]. Thus, young patients with at least 1 year of orthodontic treatment were chosen as they have higher aesthetic sensitivity. This may be due to their acquired attention to detail during orthodontic treatment [30]. Preexisting literature showed that both dentists and orthodontic patients could detect lateral incisor W/L ratio equal to or greater than 3 mm, whereas the non‐treated group could not perceive even a 4 mm change [26]. Two studies evaluated intra‐observer reliability by measuring how often participants placed duplicate images with 5% alterations adjacent to each other. A minimum Cronbach’s alpha of 70% was established. Dentists demonstrated reliability ranging from 79% to 93.3%. These findings indicate that 5% increments are consistently detectable by trained professionals [18, 23]. In contrast, laypeople exhibited greater variability in reliability (55.8%–82.9%), with some participants falling below the threshold [18, 23]. This difference in discriminatory ability accounts for the broader GP range accepted by patients (52%–77%) compared to the narrower range accepted by clinicians (62%–67%). Reduced perceptual sensitivity to subtle proportional changes increases the zone of aesthetic tolerance. Reports of repetitiveness among the participants in all groups further support this interpretation. Furthermore, similar group‐level preferences were observed with 10% increments [16], indicating that the aesthetic ranges identified in this study are robust and not dependent on the increment size.

In accordance with previous studies, our study found that orthodontists were the most critical among all dental professionals [24]. Our results align with a previous study in which 62%–67% were rated highest by the orthodontists. Florez et al. [24], while modifying a male subject’s lateral incisor perceived width dimensions (central incisor width kept constant), found orthodontists more critical, favouring 5.7:10. Lay people found the 8:10 ratio more acceptable [13]. After orthodontic treatment, central incisors and canines were closer to GP in dimensions [14, 21]. Higher aesthetic stability and increased patient satisfaction were also noted when a GP was utilised in smile makeovers [31].

Our study showed no effect of years of experience on clinicians’ ratings. This is contrary to a study by Al‐Saleh [40], stating that increased clinical experience improves aesthetic judgement. Differences in methodology between the studies may have contributed to the discrepancy as Al‐Saleh [40] used different smile photos instead of digitally altering a single photo.

This research establishes a statistically validated range of acceptable GP values, offering greater flexibility in clinical treatments. Patients and restorative dentists showed significantly broader aesthetic acceptance than orthodontists (*p*  < 0.001), reflecting their different treatment goals. Orthodontists focus on ideal occlusion using Andrews’ six keys, while restorative dentists balance the GP with the emergence profile, bilateral symmetry, and long‐term periodontal health. Defining a range for the GP supports restorative procedures such as rebuilding anomalous teeth (e.g., peg lateral incisors), restoring severely carious or worn teeth, and fabricating veneers and crowns within a zone accepted by patients. It also helps orthodontists plan rotations, derotations and achieve optimal anterior guidance within aesthetic limits. The low‐to‐moderate agreement between professions (rb = 0.257–0.396) highlights the importance of collaboration in aesthetic cases, ensuring decisions are informed by both restorative and orthodontic perspectives. A flexible range‐based GP expands finishing options for aesthetic anterior occlusions.

Our study had some limitations. There was a selection bias due to the purposive sampling method employed. The male photo had minimal buccal corridors as compared to the female photo, which had wider ones [35, 37, 38]. Clinicians found it distracting, but interestingly, none of the patients brought that up as an issue. The photos were in 2D. There is a need for 3D scans for a more realistic result. Tooth length as well as facial proportions [33, 34] play a big role in overall aesthetics, which were not covered in this study.

Based on our findings, future research should explore the interplay between facial proportions, tooth length and modified range of GP. This would help in refining the aesthetic guidelines. The usage of 3D imaging to evaluate the perception of smile aesthetics can lead to more realistic results. The research should also be directed at understanding the influence of various cultural backgrounds on aesthetic preferences. Finally, further studies should be done to better understand what shapes clinicians’ aesthetic preferences.

## 9. Conclusion

Within the limitations of this study, it was concluded that:

1. The GP is more applicable as a range rather than a fixed value. Patients preferred a broader range (52%–77%) compared to orthodontists (62%–67%), operative dentists (57%–72%), and prosthodontists (62%–72%). Kruskal–Wallis analysis confirmed significant between‐group differences for the majority of photographs (*p*  < 0.05), with significance observed across both male and female photographs, though more consistently in male lateral incisor and canine images (*p*  < 0.001).
2. Significant differences were observed between patients and all professional groups (*p*  < 0.001). Fewer differences were found among professionals, with rank‐biserial correlations indicating weak to moderate agreement (rb = 0.257–0.396).
3. Gender (*p*  > 0.05, except for two images: *p* = 0.013, *R* = 0.255) and years of clinical experience (*p*  > 0.05) had no significant effect on GP preferences.

## Funding

No funding was received for this manuscript.

## Conflicts of Interest

The authors declare no conflicts of interest.

## Data Availability

The data that support the findings of this study are available upon request from the corresponding author. The data are not publicly available due to privacy or ethical restrictions.
